# Sex-based differences in cardiovascular proteomic profiles and their associations with adverse outcomes in patients with chronic heart failure

**DOI:** 10.1186/s13293-023-00516-9

**Published:** 2023-05-17

**Authors:** Marie de Bakker, Teun B. Petersen, K. Martijn Akkerhuis, Magdalena Harakalova, Victor A. Umans, Tjeerd Germans, Kadir Caliskan, Peter D. Katsikis, Peter J. van der Spek, Navin Suthahar, Rudolf A. de Boer, Dimitris Rizopoulos, Folkert W. Asselbergs, Eric Boersma, Isabella Kardys

**Affiliations:** 1 Department of Cardiology, Erasmus MC Cardiovascular Institute, University Medical Center Rotterdam, Room Na‐316, P.O. Box 2040, 3000 CA Rotterdam, The Netherlands; 2 Department of Biostatistics, Erasmus MC, University Medical Center Rotterdam, Rotterdam, The Netherlands; 3 Department of Cardiology, Division Heart and Lungs, Circulatory Health Research Center, University Medical Center Utrecht, University of Utrecht, Utrecht, The Netherlands; 4 Regenerative Medicine Center Utrecht, University Medical Center Utrecht, University of Utrecht, Utrecht, The Netherlands; 5 Department of Cardiology, Northwest Clinics, Alkmaar, The Netherlands; 6 Department of Immunology, Erasmus MC, University Medical Center Rotterdam, Rotterdam, The Netherlands; 7 Department of Pathology, Erasmus MC, University Medical Center Rotterdam, Rotterdam, The Netherlands; 8 Department of Epidemiology, Erasmus MC, University Medical Center Rotterdam, Rotterdam, The Netherlands; 9 Amsterdam University Medical Centers, Department of Cardiology, University of Amsterdam, Amsterdam, The Netherlands; 10 Health Data Research UK and Institute of Health Informatics, University College London, London, UK

**Keywords:** Sex differences, Proteomics, Heart failure, HFrEF

## Abstract

**Background:**

Studies focusing on sex differences in circulating proteins in patients with heart failure with reduced ejection fraction (HFrEF) are scarce. Insight into sex-specific cardiovascular protein profiles and their associations with the risk of adverse outcomes may contribute to a better understanding of the pathophysiological processes involved in HFrEF. Moreover, it could provide a basis for the use of circulating protein measurements for prognostication in women and men, wherein the most relevant protein measurements are applied in each of the sexes.

**Methods:**

In 382 patients with HFrEF, we performed tri-monthly blood sampling (median follow-up: 25 [13–31] months). We selected all baseline samples and two samples closest to the primary endpoint (PEP: composite of cardiovascular death, heart transplantation, left ventricular assist device implantation, and HF hospitalization) or censoring. We then applied an aptamer-based multiplex proteomic assay identifying 1105 proteins previously associated with cardiovascular disease. We used linear regression models and gene-enrichment analysis to study sex-based differences in baseline levels. We used time-dependent Cox models to study differences in the prognostic value of serially measured proteins. All models were adjusted for the MAGGIC HF mortality risk score and *p*-values for multiple testing.

**Results:**

In 104 women and 278 men (mean age 62 and 64 years, respectively) cumulative PEP incidence at 30 months was 25% and 35%, respectively. At baseline, 55 (5%) out of the 1105 proteins were significantly different between women and men. The female protein profile was most strongly associated with extracellular matrix organization, while the male profile was dominated by regulation of cell death. The association of endothelin-1 (P_interaction_ < 0.001) and somatostatin (P_interaction_ = 0.040) with the PEP was modified by sex, independent of clinical characteristics. Endothelin-1 was more strongly associated with the PEP in men (HR 2.62 [95%CI, 1.98, 3.46], *p* < 0.001) compared to women (1.14 [1.01, 1.29], *p* = 0.036). Somatostatin was positively associated with the PEP in men (1.23 [1.10, 1.38], *p* < 0.001), but inversely associated in women (0.33 [0.12, 0.93], *p* = 0.036).

**Conclusion:**

Baseline cardiovascular protein levels differ between women and men. However, the predictive value of repeatedly measured circulating proteins does not seem to differ except for endothelin-1 and somatostatin.

**Supplementary Information:**

The online version contains supplementary material available at 10.1186/s13293-023-00516-9.

## Highlights

- Baseline levels of circulating proteins related to extracellular matrix organization were dominant in women, while those related to regulation of cell death were dominant in men.
- A significant interaction is present between sex and the circulating proteins endothelin-1 and somatostatin, in the longitudinal associations with adverse cardiovascular outcome.
- Even if circulating proteins entail similar risk in women and men, the use of the same thresholds in both sexes to ascertain the risk of future cardiovascular events may not result in equitable risk stratification, because of sex differences in baseline levels combined with underlying differences in risk of adverse events.
- Altogether, inherent sex differences in baseline levels may reflect sex differences in disease risk, suggesting that a sex-specific interpretation could be beneficial when circulating proteins are used for risk prediction in patients with chronic HF.

**Supplementary Information:**

The online version contains supplementary material available at 10.1186/s13293-023-00516-9.

## Introduction

Heart failure with reduced ejection fraction (HFrEF) is one of the most severe and prevalent manifestations of cardiovascular disease, and several aspects of this condition entail relevant sex-related differences. Although traditional HF risk factors portend a greater risk of HFrEF in women compared to men, women with HFrEF are at lower risk of HF hospitalization and mortality than men [1, 2]. Moreover, the biological response to HFrEF precursors, such as a myocardial infarction, is fundamentally different among women and men [3, 4]. The exact mechanisms underlying these differences in HFrEF risk and pathogenesis are poorly understood.

Multiple-marker assays have been developed to systematically measure extensive sets of circulating proteins that represent various biological processes [5]. Such assays offer an opportunity to elucidate differences in circulating protein profiles between women and men, which on its part, may translate into improved prognostication and ensuing therapeutic options uniquely tailored to women and men.

Sex-based differences relating to circulating proteins, which may underlie sex-based differences in disease manifestations and/or prognosis, can present themselves in several ways. The effect of a protein on disease outcome may be modified by sex. In that case, the same protein level differentially impacts in women versus men. Alternatively, a given protein may entail a similar risk of adverse outcomes in women and men, but its levels may differ between women and men. This difference in levels may then contribute to sex differences in pathogenesis and risk of adverse outcomes. Altogether, insight into sex-specific cardiovascular protein profiles and their associations with the risk of adverse outcomes may contribute to a better understanding of the pathophysiological processes involved in HF, and provide a basis for the optimal use of circulating proteins for prognostication in women and men.

In the context of HF, particularly HF with reduced ejection fraction (HFrEF), only a few studies on sex-specific circulating proteomic profiles have been carried out so far and generally focused on a limited number of proteins. For example, Suthahar et al. [6] studied ten cardiovascular disease-related biomarkers and their sex-specific associations with incident HFrEF in four community-based cohorts. Meyer et al. [7] investigated the sex-specific association of 22 biomarkers with adverse clinical outcomes in patients with HFrEF and HFpEF. Sex-based differences in protein concentration were observed in both studies, but the predictive value of cardiovascular biomarkers was similar in women and men [6, 7]. However, given the dynamic, usually progressive, nature of HF, distinguishing patients at different levels of risk of adverse events based on a single protein measurement is challenging, and serial protein measurements may contribute to dynamic risk assessment.

Therefore, we have investigated serial measurements of an elaborate set of 1,105 circulating proteins, previously associated with cardiovascular disease, in 104 women and 278 men with stable HFrEF. We aimed to assess sex-based differences in proteomic profiles and the predictive value of serially measured proteins for adverse cardiovascular events.

## Methods

### Study population

The *Serial Biomarker Measurements and New Echocardiographic Techniques in Chronic Heart Failure Patients Result in Tailored Prediction of Prognosis* (Bio-SHiFT) study is a prospective cohort study of stable patients with chronic HF (CHF), conducted in Erasmus MC, Rotterdam, and Northwest Clinics, Alkmaar, Netherlands. The study design has been described in more detail previously [8]. In brief, Bio-SHiFT enrolled patients ≥ 18 years old at the outpatient clinic, who were diagnosed with CHF ≥ 3 months before inclusion, according to the European Society of Cardiology (ESC) guidelines [9, 10]. Patients with HF hospitalization in the past three months were excluded. Study follow-up visits were predefined and scheduled every 3 months. Blood samples were collected at baseline and each follow-up visit. The routine outpatient follow-up and treatment by the treating physician continued in parallel with the study visits. The medical ethics committee of the Erasmus Medical Center in Rotterdam approved the study protocol, and all patients provided written informed consent. The study was conducted in accordance with the declaration of Helsinki and registered in ClinicalTrial.gov (NCT01851538). Between August 2011 and January 2018, a total of 398 CHF (HFrEF and HF with preserved ejection fraction) patients were enrolled. In the current investigation, 382 patients with HFrEF were evaluated.

### Baseline assessment

Information was collected on HF-related symptoms and New York Heart Association (NYHA) classification, and a physical examination was performed. Information on HF etiology, cardiovascular risk factors, medical history, and treatment was retrieved primarily from hospital records and was checked in case of ambiguities.

### Sample collection and processing

Within 2 h after collection, blood samples were processed, and EDTA plasma was stored at − 80 °C. Accordingly, at the time of the outpatient visits, results of the proteomic analysis were not available to treating physicians. Laboratory personnel was blinded for clinical data and patient outcomes. For the current investigation, all baseline blood samples were selected. Additionally, samples from the tri-monthly follow-up visits were used. Specifically, the last two samples drawn before the primary endpoint were selected, or the last two samples that were available before censoring in patients who remained endpoint-free (visualized in Additional file 1: Fig S1). In total, 1,070 samples during a median [25th–75th percentile] follow-up of 25 [13–31] months were available for the current study. Previous investigations using all available samples in our patient cohort have demonstrated that the concentrations of several plasma and urine biomarker candidates change in the months preceding the occurrence of an adverse event [8, 11]. By selecting the last two samples prior to the incident study endpoint, we aimed to capture these changes while improving efficiency.

### Proteomic analysis

The aptamer-based proteomic SOMAscan assay (Somalogic, Boulder, Colorado, United States) was used to measure 5284 plasma proteins as previously described [12]. SOMAscan utilizes single-stranded DNA-based protein affinity reagents called SOMAmers (Slow Off-rate Modified Aptamers). SOMAmers bind proteins with high specificity and affinity, and slow dissociation rates, minimizing nonspecific binding interactions. Somalogic’s previously described standard processes for normalization, calibration, and quality control were followed (see Additional file 1: Methods for details) [13]. The readout of the SOMAscan assay is in normalized relative fluorescent units (RFUs). These intensities are directly related to the amount of available epitope of the target protein in the original sample. Previous studies reported high assay reproducibility and low technical variability of SOMAscan [14, 15].

For the current investigation, the subset of proteins associated with cardiovascular functions or diseases according to Ingenuity Pathway Analysis (IPA) was used [16]. IPA is a resource that associates lists of proteins with biological pathways, functions, and diseases based on a large database of existing literature. Thus, out of the total 5284 modified aptamers, aptamers against 1105 proteins associated with cardiovascular (patho)physiology were included in the current analyses. Individual sample quality was judged by comparing the normalized median signal relative to the external reference standard. Data from 1066 samples passed quality control criteria.

### Clinical study endpoints

A clinical event committee reviewed hospital records and discharge letters and adjudicated the study endpoints. The primary endpoint comprised the composite of cardiovascular death, heart transplantation (HTx), left ventricular assist device (LVAD) implantation, and hospitalization for the management of acute or worsened HF. In patients who reached multiple endpoints, only the first was used for analysis. Hospitalization for acute or worsened HF was defined as hospitalization for an exacerbation of HF symptoms in combination with two of the following: brain natriuretic peptide or N-terminal-pro hormone B-type natriuretic peptide (NT‐proBNP) > 3 × normal upper limit, signs of worsening HF, such as pulmonary rales, raised jugular venous pressure or peripheral edema, increased dose or intravenous administration of diuretics, and/or administration of positive inotropic agents [9].

### Statistical analysis

Continuous variables are presented as mean, standard deviation (SD), or median, 25th to 75th percentile, as appropriate. Categorical variables are presented as absolute numbers (%). Differences in clinical characteristics between men and women were assessed by Student’s *t*-tests or Mann–Whitney-*U* tests, as appropriate. The Chi-squared test or Fisher’s exact test, as appropriate, was used for the comparison of proportions. For the following analyses, protein levels were log2-transformed to achieve normal distributions.

Linear regression using only baseline samples was applied to reveal sex-based differences in mean protein baseline levels, while adjusting for the MAGGIC HF mortality risk score [17]. *p*-values were corrected for multiple testing using the Benjamini–Hochberg method (FDR < 0.05). Gene-enrichment analysis was performed using ToppGene Suite [18] for the proteins that remained significantly different between women and men. The overrepresentation analysis was performed using Gene Ontology (GO) processes, providing a computational representation of biological processes enriched in the set of significant proteins against all cardiovascular disease-related proteins on the SOMAscan assay.

Cumulative incidence of the primary endpoint was studied by the method of Kaplan–Meier, and differences between women and men were evaluated by the log-rank test.

Sex-based differences in the prognostic value of serially measured circulating proteins were evaluated using time-dependent Cox models for each individual protein. Values of the repeatedly measured circulating proteins were estimated and extracted for the moments at which the proteins were actually measured by linear mixed effects (LME) modeling (see Additional file 1: Methods for details). Subsequently, the estimated protein levels were standardized and entered into single-protein time-dependent Cox models, together with sex and an interaction term for sex and protein level. Multiplicative interaction (signifying that the combined effect of sex and protein level is larger [or smaller] than the product of the individual effects, visualized in Additional file 1: Fig S2A) was assessed using the regression coefficient of the interaction term. Additive interaction (signifying that the combined effect of sex and protein level is larger [or smaller] than the sum of the individual effects, visualized in Additional file 1: Fig S2B) was assessed using relative excess risk due to interaction (RERI) and the delta method [19]. *p*-values were corrected for multiple testing using the Benjamini–Hochberg method (FDR < 0.05).

Data analyses are performed using R (version 4.1.2.), in particular the packages nlme and survival. A two-sided *p*-value < 0.05 or FDR < 0.05 was considered statistically significant, depending on the context.

## Results

### Clinical characteristics of study population

In total, 104 (27.2%) women and 278 (72.8%) men were included, who had similar mean age (62 ± 13 versus 64 ± 13, respectively, *p* = 0.138) (Table 1). Women had a significantly lower body mass index (26.2 ± 4.6 versus 27.6 ± 4.4, *p* = 0.007) and were more often current smokers (13.5 versus 8.3%, *p* = 0.016) than men. Women less often had ischemic etiology of HF compared to men (26.9 versus 49.6%, *p* < 0.001). Moreover, the prevalence of comorbidities, such as the history of myocardial infarction (24.5 versus 43.6%, *p* = 0.001) or percutaneous coronary intervention (20.2 versus 37.8%, *p* = 0.002), atrial fibrillation (26.2 versus 40.1%, *p* = 0.017), and known hypercholesterolemia (32.7 versus 46.8%, *p* = 0.018), was also lower in women, as were median baseline levels of high-sensitivity troponin T (13.5 [7.4, 27.6] versus 20.0 [12.0, 39.0], *p* < 0.001). No clinically relevant differences in mean left ventricular ejection fraction (women: 31 ± 11 versus men: 29 ± 10, *p* = 0.164), NT-proBNP (women: 128.2 [53.6, 262.2] versus men: 165.0 [58.0, 292.9], *p* = 0.396) or C-reactive protein (CRP) (women: 2.2 [0.9, 4.6] versus men: 2.0 [1.0, 4.7], *p* = 0.633) were present between sexes.

**Table 1:** Baseline characteristics of study population

Demographics	Total population	Women	Men	*p*-value
	*n* = 382	*n* = 104	*n* = 278	
Age [mean (SD)]	63.3 (13.1)	61.6 (13.4)	63.9 (13.0)	0.138
Caucasian ethnicity (%)	351 (92.6)	96 (94.1)	255 (92.1)	0.647
**Clinical characteristics**				
Body mass index, kg/m^2^ [mean (SD)]	27.2 (4.5)	26.2 (4.6)	27.6 (4.4)	**0.007**
Systolic blood pressure, mmHg [mean (SD)]	115.3 (21.3)	115.2 (22.2)	115.3 (21.0)	0.993
Diastolic blood pressure, mmHg [mean (SD)]	70.0 (10.5)	70.1 (10.8)	69.9 (10.5)	0.900
**Features of heart failure**				
Duration of HF, years ǂ	4.2 [1.6, 9.5]	3.7 [1.3, 7.8]	4.4 [1.7, 9.8]	0.178
NYHA class (%)				0.740
NYHA class I	94 (24.7)	25 (24.0)	69 (25.0)	
NYHA class II	182 (47.9)	53 (51.0)	129 (46.7)	
NYHA class III and IV	104 (27.4)	26 (25.0)	78 (28.3)	
LVEF [mean (SD)]*	29.8 (10.3)	31.2 (10.7)	29.3 (10.1)	0.164
**Heart failure etiology**				
Ischemic heart disease (% yes)	166 (43.5)	28 (26.9)	138 (49.6)	**< 0.001**
Cardiomyopathy (% yes)	122 (31.9)	37 (35.6)	85 (30.6)	0.418
Hypertension (% yes)	33 (8.6)	13 (12.5)	20 (7.2)	0.150
Secondary to valvular heart disease (% yes)	12 (3.1)	5 (4.8)	7 (2.5)	0.321
Other etiology (% yes)	26 (6.8)	11 (10.6)	15 (5.4)	0.118
Unknown etiology (% yes)	27 (7.1)	10 (9.6)	14 (5.0)	0.160
**Established biomarker levels**				
NT-proBNP (pmol/L)^ǂ^	145.0 [54.7, 289.0]	128.2 [53.6, 262.2]	165.0 [58.0, 292.9]	0.396
Hs-TnT (ng/L)^ǂ^	18.0 [10.3, 34.0]	13.5 [7.4, 27.6]	20.0 [12.0, 39.0]	**< 0.001**
CRP (mg/L)^ǂ^	2.0 [0.9, 4.7]	2.2 [0.9, 4.6]	2.0 [1.0, 4.7]	0.633
**Medical history**				
Myocardial infarction (% yes)	145 (38.5)	25 (24.5)	120 (43.6)	**0.001**
PCI (% yes)	126 (33.0)	21 (20.2)	105 (37.8)	**0.002**
CABG (% yes)	54 (14.1)	7 (6.7)	47 (16.9)	**0.018**
Atrial fibrillation (% yes)	137 (36.3)	27 (26.2)	110 (40.1)	**0.017**
CRT (% yes)	113 (29.7)	28 (27.2)	85 (30.6)	0.605
Pacemaker (% yes)	85 (23.0)	22 (21.8)	63 (23.5)	0.832
Chronic renal failure (% yes)	181 (47.6)	52 (50.0)	129 (46.7)	0.651
Diabetes mellitus (% yes)	98 (25.7)	26 (25.0)	72 (25.9)	0.962
Hypercholesterolemia (% yes)	160 (42.9)	34 (32.7)	126 (46.8)	**0.018**
COPD (% yes)	50 (13.3)	12 (11.9)	38 (13.8)	0.750
**Intoxications**				
Smoking (%)				**0.016**
Never	109 (28.7)	38 (36.5)	71 (25.7)	
Current	37 (9.7)	14 (13.5)	23 (8.3)	
Former (> 30 days)	234 (61.6)	52 (50.0)	182 (65.9)	
**Medication use**				
Beta blockers (% yes)	350 (91.9)	94 (90.4)	256 (92.4)	0.662
ACE-I (% yes)	258 (67.7)	72 (69.2)	186 (67.1)	0.792
ARB (% yes)	107 (28.0)	29 (27.9)	78 (28.1)	1.000
Aldosterone antagonist (% yes)	293 (76.7)	82 (78.8)	211 (75.9)	0.638
Loop diuretics (% yes)	353 (92.4)	98 (94.2)	255 (91.7)	0.545
Thiazide diuretics (% yes)	12 (3.1)	1 (1.0)	11 (4.0)	0.193
Aspirin (% yes)	77 (20.2)	21 (20.2)	56 (20.2)	1.000
Anticoagulants (% yes)	279 (73.0)	71 (68.3)	208 (74.8)	0.248
**MAGGIC risk score**				
MAGGIC risk score [mean (SD)]	20.3 (7.2)	18.4 (7.8)	21.0 (6.8)	0.001

*ACE* angiotensin-converting enzyme, *ARB* angiotensin receptor blocker, *CABG* coronary artery bypass graft, *CRP* C-reactive protein, *CRT* cardiac resynchronization therapy, *COPD* chronic obstructive pulmonary disease, *hs-TnT* high-sensitivity troponin T, *LVEF* left ventricular ejection fraction, *NT-proBNP* N-terminal-pro hormone B-type natriuretic peptide, *NYHA* New York Heart Association, *PCI* percutaneous coronary intervention, *SD* standard deviation

^ǂ^All biomarker levels and duration of heart failure are presented as median [25th–75th percentile]. *Missing for 81 patients

A *p*-value < 0.05 is considered statistically significant

### Sex-based differences in cardiovascular proteomic profile at baseline

**Fig. 1:**
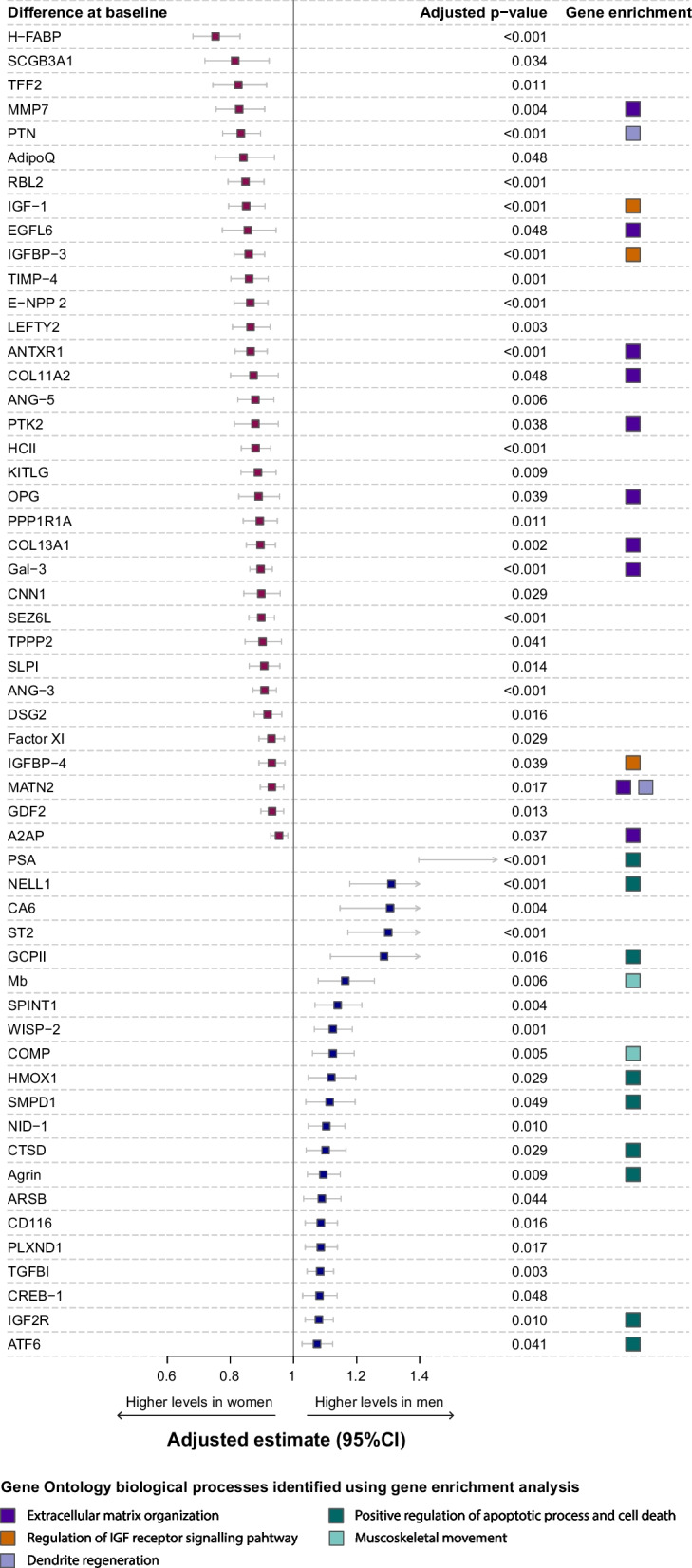
Sex-based differences in protein level at baseline. The mean difference (i.e., the regression coefficient for sex) is depicted for proteins showing a statistically significant difference (FDR < 0.05) between women and men at baseline. Analyses were adjusted for the MAGGIC risk score. The over-represented proteins in the Gene Ontology biological processes, identified using the gene-enrichment analysis, are highlighted in the column on the right

After correction for multiple testing, 55 proteins showed statistically significant differences in circulating protein levels between women and men at baseline (Fig. 1). Specifically, 34 proteins showed higher mean levels in women, including, for example, heart-type fatty acid binding protein (H-FABP), adiponectin (AdipoQ), osteoprotegerin (OPG), and galectin-3 (Gal-3), while mean levels of 21 proteins were higher in men, including for example, prostate-specific antigen (PSA), interleukin 1 receptor-like 1 (ST2), myoglobin (Mb), and transforming growth factor β1 (TGFB1).

The five biological processes that were most strongly associated with the female circulating protein profile were related to extracellular matrix organization (GO:0030198, GO:0043062, and GO:0045229), regulation of the insulin-like growth factor receptor signaling pathway (GO:0043568) and dendrite regeneration (GO:0031104), while the five processes that dominated the male profile were related to positive regulation of apoptotic processes and cell death (GO:0043065, GO:0043068, and GO:0010942) and musculoskeletal movement (GO:0050881 and GO:0050879), irrespectively of primary endpoint status (Fig. 1 and Table 2).

**Table 2:** Biological processes associated with sex-specific protein profiles

A. Top 5 enriched biological processes in women*			
ID	Name	*p*-value	Genes [# selected genes / # in annotation]
GO:0030198	Extracellular matrix organization	< 0.001	[10/107]: MMP7, EGFL6, ANTXR1, COL11A2, PTK2, TNFRSF11B, COL13A1, Gal-3, MATN2, A2AP
GO:0043062	Extracellular structure organization	< 0.001	[10/107]: MMP7, EGFL6, ANTXR1, COL11A2, PTK2, TNFRSF11B, COL13A1, Gal-3, MATN2, A2AP
GO:0045229	External encapsulating structure organization	< 0.001	[10/107]: MMP7, EGFL6, ANTXR1, COL11A2, PTK2, TNFRSF11B, COL13A1, Gal-3, MATN2, A2AP
GO:0043568	Positive regulation of insulin-like growth factor receptor signaling pathway	< 0.001	[3/7]: IGF-1, IGFBP-3, IGFBP-4
GO:0031104	Dendrite regeneration	0.003	[2/3]: PTN, MATN2

B. Top 5 enriched biological processes in men*			
ID	Name	*p*-value	Genes [# in selection / # in annotation]
GO:0043065	Positive regulation of apoptotic process	0.002	[9/164]: PSA, NELL1, GCPII, HMOX1, SMPD1, CTSD, Agrin, IGF2R, ATF6
GO:0043068	Positive regulation of programmed cell death	0.002	[9/166]: PSA, NELL1, GCPII, HMOX1, SMPD1, CTSD, Agrin, IGF2R, ATF6
GO:0010942	Positive regulation of cell death	0.004	[9/182]: PSA, NELL1, GCPII, HMOX1, SMPD1, CTSD, Agrin, IGF2R, ATF6
GO:0050881	Musculoskeletal movement	0.001	[2/9]: Mb, COMP
GO:0050879	Multicellular organismal movement	0.001	[2/9]: Mb, COMP

*Gene-enrichment analysis was performed using ToppGene Suite for the proteins that remained significantly different between women and men. The overrepresentation analysis was performed using Gene Ontology (GO) processes, providing a computational representation of biological processes enriched in the set of significant proteins against all cardiovascular disease-related proteins on the SOMAscan assay. Additional file 1: Fig S3 shows a graphical representation of other biological processes, beyond the top 5, associated with the sex-specific protein profiles

Graphical summaries of all biological processes (beyond the ‘top 5’) associated with the circulating protein profiles in women and men are provided in Additional file 1: Fig S3. For women, this summary highlights processes related to positive regulation of insulin-like growth factor receptor signaling pathway and negative regulation of synapse organization. For men, this summary highlights processes associated with positive regulation of apoptotic processes and protein prenylation.

### Sex-based differences in the prognostic value of serially measured circulating proteins

**Fig. 2:**
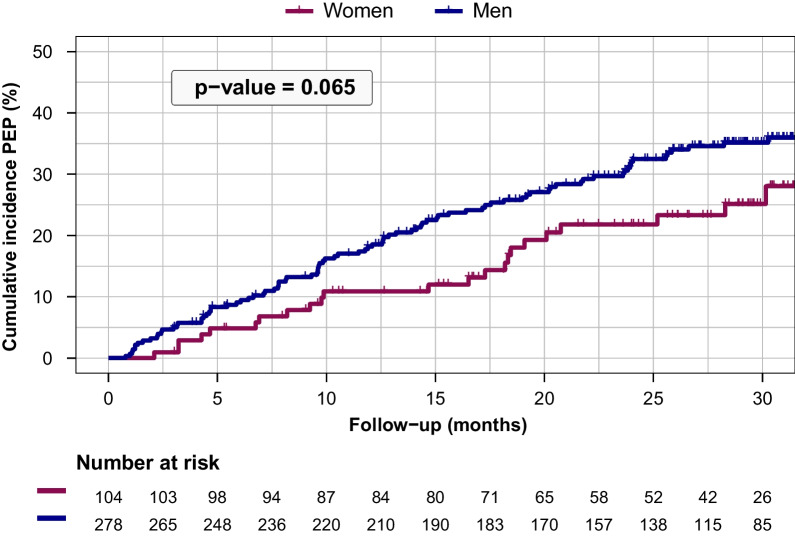
Primary endpoint-free survival probability in women and men. The Kaplan–Meier estimate of the survival function for the primary endpoint in women (red) and men (blue)

During a median [25th–75th percentile] follow-up of 25 [13–31] months, a total of 23 women and 91 men reached the primary endpoint (Additional file 1: Table S1). Women had a lower cumulative incidence of the primary endpoint during follow-up compared to men (25% versus 35%, respectively, at 30 months), although this difference did not reach statistical significance (*p* = 0.065, Fig. 2).

**Fig. 3:**
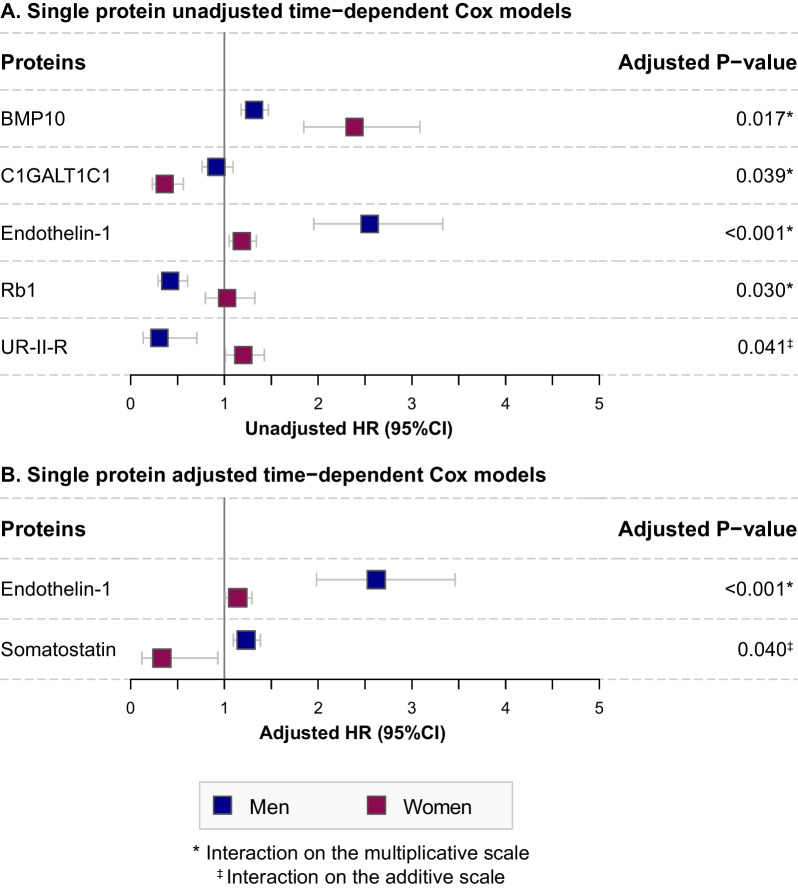
Sex-based differences in the predictive value of serially measured proteins. The estimated hazard ratios for serially measured proteins showing a statistically significant (FDR < 0.05) interaction with sex on the multiplicative scale or the additive scale are depicted for men (blue) and women (red) separately. Analyses were unadjusted (**A**) or adjusted for the MAGGIC risk score (**B**)

Bone morphogenetic protein 10 (BMP10), C1GALT1 specific chaperone 1 (C1GALT1C1), endothelin-1, and retinoblastoma 1 (Rb1) showed a statistically significant interaction with sex on the multiplicative scale in single-protein, unadjusted models (Fig. 3 and Additional file 1: Fig S4). After correction for the MAGGIC risk score, the interaction of endothelin-1 with sex remained statically significant (HR_interaction term_ [95%CI]: 2.29 [1.69–3.11], *p* < 0.001), implying that with each unit increase of the circulating protein, the risk of having a primary endpoint in men (HR [95%CI]: 2.62 [1.98–3.46], *p* < 0.001) is 2.29 times the risk associated with each unit increase of the circulating protein in women (HR [95%CI]: 1.14 [1.01–1.29], *p* = 0.036). In other words, the combined effect of sex and endothelin-1 level is larger than the product of the individual effects.

Urotensin II receptor (UR-II-R) showed a statistically significant interaction with sex on the additive scale (RERI [95%CI]: − 1.13 [− 1.66 – − 0.59], *p* = 0.041) (Fig. 3 and Additional file 1: Fig S4). After correction for the MAGGIC risk score, only somatostatin showed a statistically significant interaction on the additive scale (RERI [95%CI]: 1.02 [0.54–1.50], *p* = 0.040). This implies that the hazard ratio of the primary endpoint in men is 1.02 larger with each unit increase in circulating protein level than if there was no interaction between sex and protein level. Moreover, somatostatin level was positively associated with the primary endpoint in men (HR [95%CI]: 1.23 [1.10–1.38], *p* < 0.001), while an inverse association was found in women (HR [95%CI]: 0.33 [0.12–0.93], *p* = 0.036). In other words, the combined effect of sex and somatostatin level is larger than the sum of the individual effects.

## Discussion

We conducted a sex-specific analysis of comprehensive cardiovascular proteomic profiles and their associations with clinical outcome in patients with HFrEF. Our study has two main findings. First, women and men show differences in baseline levels of circulating proteins related to extracellular matrix organization and regulation of cell death. Second, a significant interaction is present between sex and the circulating proteins endothelin-1 and somatostatin, in the longitudinal associations with adverse cardiovascular outcome. These findings suggest that a sex-specific risk assessment approach could be beneficial when circulating proteins are used for risk prediction in patients with chronic HF.

Our study has several strengths. First, our study is the first that conducted a sex-specific analysis of such an elaborate cardiovascular proteomic profile of patients with chronic HFrEF. Second, our highly frequent blood sampling design enabled us to account for the temporal changes and dynamic nature of HF and to study the association of repeatedly measured circulating proteins with clinical outcomes during follow-up. So far, studies on the sex-specific associations of circulating proteins in the context of HF have usually examined limited numbers (one or a few) of biomarkers and traditionally performed cross-sectional measurements only and related them to adverse events occurring over many years thereafter [6, 7, 20, 21].

Previous studies on sex differences in circulating proteins in HF are limited in number but have already suggested sex differences in the absolute concentrations of established biomarkers [22, 23]. For example, levels of NT-proBNP are often higher in women than in men with chronic HF, whereas levels of ST2 are lower in women [22, 23]. We extend current knowledge by evaluating an elaborate set of 1,105 plasma proteins to identify sex-based differences in protein levels and in associated cardiovascular-related pathophysiological processes within various organ systems, as reflected in the circulation. We observed that circulating proteins associated with extracellular matrix organization were overrepresented in women, while circulating proteins reflecting apoptotic processes were overrepresented in men. Since HF affects many tissues and organs throughout the body, the concentrations of circulating proteins in patients with HF also reflect production in stressed non-cardiac tissues, either as a consequence of the failing heart or other underlying comorbidities [24, 25]. The observed differences between women and men may be attributed to the role of sex hormones or sex hormone receptors [26], the presence of extracellular matrix organization- or apoptosis-related genes on the sex chromosomes [27] and/or sex differences in cardiovascular epigenetics [28], although the exact mechanisms are not completely understood. Moreover, it should be kept in mind that the baseline sex-related differences observed in the current study do not need to indicate sex-specific pathophysiology but may also be a manifestation of physiological sex-based differences.

Although previous studies have shown differences in absolute levels of proteins between men and women, reports on the sex-specific predictive value in patients with HF are limited [7, 21, 29–31]. Baseline NT-proBNP more strongly predicted all-cause mortality in men than in women with HF [7, 30], while high-sensitivity troponin T (hs-TnT) and hs-TnI showed similar predictive value for both sexes in patients with HFrEF [31]. In our recent investigation of the temporal patterns of NT-proBNP, hs-TnT, and CRP, as measured in the first inclusion round of the Bio-SHiFT study, the association with adverse clinical events appeared to be more prominent in women than in men with HFrEF [29]. In contrast, strikingly similar associations of most circulating proteins with clinical outcomes of HF were found for both sexes in the current study. These findings are in line with recent studies by Raafs et al. [20] and Suthahar et al. [6], which reported no statistically significant differences in the associations of 252 and ten circulating proteins, respectively, with incident HF between women and men. Nonetheless, we did observe sex-related differences in the associations of repeatedly measured endothelin-1 and somatostatin with the primary endpoint. Endothelin-1 was more strongly associated with the primary endpoint in men than in women. Endothelin-1 is considered a predictor of adverse clinical outcomes in HF and plays a key role in many aspects of cardiac physiology and pathology, such as hypertension, cardiac contractility, and cardiac remodeling [32, 33]. Sex-related differences have been reported in receptor expression and vascular response to the endothelin-1 receptors [33], which may contribute to the sex-specific association observed in the current study. Somatostatin, also known as growth hormone inhibiting hormone, is known for its strong regulatory effects throughout the body, such as suppression of insulin-like growth factor I, growth hormone, and insulin. In the current study, somatostatin was positively associated with the primary endpoint in men, but inversely associated in women. Previous studies have shown that somatostatin exerts a cardioprotective effect in in vitro and animal models of ischemia/reperfusion injury [34], whereas increased concentrations were associated with a higher mortality risk in patients with advanced HF[35]. Higher circulating somatostatin levels have been linked to male sex in the general population [36]. The sex-specific role of somatostatin in the pathophysiology of HF remains unknown.

### Perspectives and significance

Although in the current study few sex differences were present in the associations of the circulating proteins with clinical outcomes of HF, the differences that we found in the protein profiles themselves warrant attention. In clinical practice, for risk assessment, often uniform thresholds are proposed for circulating proteins. For example, a ST2 threshold of 35 ng/ml is recommended in both women and men [37]. On the other hand, previous literature has shown that women with HFrEF have lower risk of adverse events than men of the same age [1]. This tendency was also confirmed in the current study. Altogether our findings imply that, if all other risk factors are kept constant, women with elevated proteins levels above the threshold, will have lower absolute risk of adverse events than their male counterparts. In other words, if a uniform protein threshold is applied, women will likely only reach the same absolute risk of adverse events as men if they have more concomitant risk factors. Since the clinical consequences of ‘high’ protein levels will thus be different for men and women, a sex-specific interpretation is warranted when using such an approach to circulating proteins for risk stratification. Alternatively, the approach could be personalized, and circulating proteins could be used as continuous, longitudinal measures within a dynamic risk prediction tool that also incorporates other clinical features, thereby enabling appropriate risk assessment for all relevant patients subgroups. The clinical and economic consequences of incorporating sex-specific protein profiles in clinical practice warrant further research.

Some limitations need to be acknowledged. First, men were overrepresented in the Bio-SHiFT study. Second, SOMAmer reagents are selected against proteins in their native folded conformations. Hence, unfolded and denatured proteins are not detected. Moreover, the SOMAscan assay does not provide absolute concentrations but RFUs. While these values can be used for comparing women and men, the absolute concentrations based on validated assays (e.g., ELISA) are recommended for clinical applications. Third, we assessed sex-based differences in circulating proteins previously associated with cardiovascular disease. Investigating a wider range of proteins was beyond the scope of the current study. Finally, the Bio-SHiFT study comprises a mostly white population and generalizing our findings to other ethnic groups should be performed with caution.

## Conclusion

In conclusion, although baseline cardiovascular protein levels differ between women and men, the predictive value of repeatedly measured circulating proteins does not seem to differ. Nevertheless, the association with adverse cardiovascular outcome of endothelin-1 and somatostatin, related to hypertension and hormone regulation, respectively, was modified by sex in the current study. Further investigation into sex-based differences in proteomic profiles may provide mechanistic insight into sex differences in HF pathogenesis.

## Supplementary Information

**Additional file 1.** Supplemental materials.

## Data Availability

Anonymized data that support the findings of this study will be made available to other researchers for the purposes of reproducing the results upon reasonable request and in accordance with a data-sharing agreement.

## References

[1] Dewan P, Rørth R, Jhund PS, Shen L, Raparelli V, Petrie MC (2019). Differential impact of heart failure with reduced ejection fraction on men and women. J Am Coll Cardiol.

[2] Sillars A, Ho FK, Pell GP, Gill JMR, Sattar N, Gray S (2020). Sex differences in the association of risk factors for heart failure incidence and mortality. Heart.

[3] Swaraj S, Kozor R, Arnott C, Di Bartolo BA, Figtree GA (2021). Heart failure with reduced ejection fraction—does sex matter?. Curr Heart Failure Reports..

[4] Kessler EL, Rivaud MR, Vos MA, van Veen TAB (2019). Sex-specific influence on cardiac structural remodeling and therapy in cardiovascular disease. Biol Sex Differ.

[5] Smith JG, Gerszten RE (2017). Emerging affinity-based proteomic technologies for large-scale plasma profiling in cardiovascular disease. Circulation.

[6] Suthahar N, Lau ES, Blaha MJ, Paniagua SM, Larson MG, Psaty BM (2020). Sex-specific associations of cardiovascular risk factors and biomarkers with incident heart failure. J Am Coll Cardiol.

[7] Meyer S, van der Meer P, van Deursen VM, Jaarsma T, van Veldhuisen DJ, van der Wal MH (2013). Neurohormonal and clinical sex differences in heart failure. Eur Heart J.

[8] van Boven N, Battes LC, Akkerhuis KM, Rizopoulos D, Caliskan K, Anroedh SS (2018). Toward personalized risk assessment in patients with chronic heart failure: detailed temporal patterns of NT-proBNP, troponin T, and CRP in the Bio-SHiFT study. Am Heart J.

[9] McMurray JJ, Adamopoulos S, Anker SD, Auricchio A, Böhm M, Dickstein K (2012). ESC Guidelines for the diagnosis and treatment of acute and chronic heart failure 2012: The Task Force for the Diagnosis and Treatment of Acute and Chronic Heart Failure 2012 of the European Society of Cardiology. Developed in collaboration with the Heart Failure Association (HFA) of the ESC. Eur Heart J.

[10] Paulus WJ, Tschöpe C, Sanderson JE, Rusconi C, Flachskampf FA, Rademakers FE (2007). How to diagnose diastolic heart failure: a consensus statement on the diagnosis of heart failure with normal left ventricular ejection fraction by the Heart Failure and Echocardiography Associations of the European Society of Cardiology. Eur Heart J.

[11] Brankovic M, Akkerhuis KM, van Boven N, Anroedh S, Constantinescu A, Caliskan K (2018). Patient-specific evolution of renal function in chronic heart failure patients dynamically predicts clinical outcome in the Bio-SHiFT study. Kidney Int.

[12] Gold L, Ayers D, Bertino J, Bock C, Bock A, Brody EN (2010). Aptamer-based multiplexed proteomic technology for biomarker discovery. PLoS ONE.

[13] Williams SA, Kivimaki M, Langenberg C, Hingorani AD, Casas JP, Bouchard C (2019). Plasma protein patterns as comprehensive indicators of health. Nat Med.

[14] Kim CH, Tworoger SS, Stampfer MJ, Dillon ST, Gu X, Sawyer SJ (2018). Stability and reproducibility of proteomic profiles measured with an aptamer-based platform. Sci Rep.

[15] Candia J, Cheung F, Kotliarov Y, Fantoni G, Sellers B, Griesman T (2017). Assessment of variability in the SOMAscan assay. Sci Rep.

[16] Krämer A, Green J, Pollard J, Tugendreich S (2014). Causal analysis approaches in ingenuity pathway analysis. Bioinformatics.

[17] Pocock SJ, Ariti CA, McMurray JJ, Maggioni A, Køber L, Squire IB (2013). Predicting survival in heart failure: a risk score based on 39 372 patients from 30 studies. Eur Heart J.

[18] Chen J, Bardes EE, Aronow BJ, Jegga AG (2009). ToppGene Suite for gene list enrichment analysis and candidate gene prioritization. Nucleic Acids Res.

[19] Hosmer DW, Lemeshow S (1992). Confidence interval estimation of interaction. Epidemiology.

[20] Raafs A, Verdonschot J, Ferreira JP, Wang P, Collier T, Henkens M (2021). Identification of sex-specific biomarkers predicting new-onset heart failure. ESC Heart Fail.

[21] Stienen S, Ferreira JP, Kobayashi M, Preud'homme G, Dobre D, Machu JL (2020). Sex differences in circulating proteins in heart failure with preserved ejection fraction. Biol Sex Differ.

[22] Cediel G, Codina P, Spitaleri G, Domingo M, Santiago-Vacas E, Lupón J, et al. Gender-related differences in heart failure biomarkers. Front Cardiovasc Med. 2021;7 10.3389/fcvm.2020.617705 PMC7813809 33469552

[23] Suthahar N, Meems LMG, Ho JE, de Boer RA (2020). Sex-related differences in contemporary biomarkers for heart failure: a review. Eur J Heart Fail.

[24] Piek A, Du W, de Boer RA, Silljé HHW (2018). Novel heart failure biomarkers: why do we fail to exploit their potential?. Crit Rev Clin Lab Sci.

[25] Du W, Piek A, Schouten EM, van de Kolk CWA, Mueller C, Mebazaa A (2018). Plasma levels of heart failure biomarkers are primarily a reflection of extracardiac production. Theranostics.

[26] Iorga A, Cunningham CM, Moazeni S, Ruffenach G, Umar S, Eghbali M (2017). The protective role of estrogen and estrogen receptors in cardiovascular disease and the controversial use of estrogen therapy. Biol Sex Differ.

[27] Winham SJ, de Andrade M, Miller VM (2015). Genetics of cardiovascular disease: Importance of sex and ethnicity. Atherosclerosis.

[28] Hartman RJG, Huisman SE, den Ruijter HM (2018). Sex differences in cardiovascular epigenetics-a systematic review. Biol Sex Differ.

[29] Schreuder MM, Schuurman A, Akkerhuis KM, Constantinescu AA, Caliskan K, van Ramshorst J (2021). Sex-specific temporal evolution of circulating biomarkers in patients with chronic heart failure with reduced ejection fraction. Int J Cardiol.

[30] Vergaro G, Gentile F, Aimo A, Januzzi JL, Richards AM, Lam CSP (2022). Circulating levels and prognostic cut-offs of sST2, hs-cTnT, and NT-proBNP in women vs. men with chronic heart failure. ESC Heart Failure..

[31] Gohar A, Chong JPC, Liew OW, den Ruijter H, de Kleijn DPV, Sim D (2017). The prognostic value of highly sensitive cardiac troponin assays for adverse events in men and women with stable heart failure and a preserved vs. reduced ejection fraction. Eur J Heart Failure..

[32] Jankowski W, Lagassé HAD, Chang WC, McGill J, Jankowska KI, Gelinas AD (2020). Modified aptamers as reagents to characterize recombinant human erythropoietin products. Sci Rep.

[33] Gohar EY, Giachini FR, Pollock DM, Tostes RC (2016). Role of the endothelin system in sexual dimorphism in cardiovascular and renal diseases. Life Sci.

[34] Vörös I, Sághy É, Pohóczky K, Makkos A, Onódi Z, Brenner GB (2021). Somatostatin and its receptors in myocardial ischemia/reperfusion injury and cardioprotection. Front Pharmacol.

[35] Deis T, Balling L, Rossing K, Boesgaard S, Kistorp CM, Wolsk E (2020). Plasma somatostatin in advanced heart failure: association with cardiac filling pressures and outcome. Cardiology.

[36] Abbasi A, Kieneker LM, Corpeleijn E, Gansevoort RT, Gans RO, Struck J (2017). Plasma N-terminal Prosomatostatin and Risk of Incident Cardiovascular Disease and All-Cause Mortality in a Prospective Observational Cohort: the PREVEND Study. Clin Chem.

[37] Januzzi JL, Mebazaa A, Di Somma S (2015). ST2 and prognosis in acutely decompensated heart failure: the International ST2 consensus panel.. Am J Cardiol.

[38] Supek F, Bošnjak M, Škunca N, Šmuc T (2011). REVIGO summarizes and visualizes long lists of gene ontology terms. PLoS ONE.

