# Supplementary material for: Sex-based differences in cardiovascular proteomic profiles and their associations with adverse outcomes in patients with chronic heart failure
Source: Biol Sex Differ. 2023 May 17;14:29. doi: 10.1186/s13293-023-00516-9 (PMC10193800; doi:10.1186/s13293-023-00516-9)
Supplement: Supplementary file 1 — Additional file 1. Supplemental materials. [file 13293_2023_516_MOESM1_ESM.pdf]

SUPPLEMENTAL MATERIAL

Sex-based differences in cardiovascular proteomic profiles and their associations with adverse outcome in patients with chronic heart failure

Running title: Sex differences in heart failure biomarkers

Marie de Bakker MSc[1], Teun B. Petersen MSc[1,2], K. Martijn Akkerhuis MD PhD[1], Magdalena Harakalova, MD PhD[3,4], Victor A. Umans MD PhD[5], Tjeerd Germans MD PhD[5], Kadir Caliskan MD PhD[1], Peter D. Katsikis MD PhD[6], Peter J. van der Spek PhD[7], Navin Suthahar MD MSc PhD[1], Rudolf A de Boer MD PhD[1], Dimitris Rizopoulos PhD[2,8], Folkert W. Asselbergs MD PhD[9,10], Eric Boersma PhD[1], Isabella Kardys MD PhD[1]

[1] *Department of Cardiology, Erasmus MC Cardiovascular Institute, University Medical Center Rotterdam, Rotterdam, the Netherlands*
[2] *Department of Biostatistics, Erasmus MC, University Medical Center Rotterdam, Rotterdam, the Netherlands.*
[3] *Department of Cardiology, Division Heart and Lungs, University Medical Center Utrecht, University of Utrecht, Utrecht, the Netherlands.*
[4] *Regenerative Medicine Center Utrecht, University Medical Center Utrecht, University of Utrecht, Utrecht, the Netherlands*
[5] *Department of Cardiology, Northwest Clinics, Alkmaar, the Netherlands.*
[6] *Department of Immunology, Erasmus MC, University Medical Center Rotterdam, Rotterdam, the Netherlands.*
[7] *Department of Pathology, Erasmus MC, University Medical Center Rotterdam, Rotterdam, the Netherlands.*
[8] *Department of Epidemiology, Erasmus MC, University Medical Center Rotterdam, Rotterdam, the Netherlands.*
[9] *Amsterdam University Medical Centers, Department of Cardiology, University of Amsterdam, Amsterdam, The Netherlands*
[10] *Health Data Research UK and Institute of Health Informatics, University College London, London, United Kingdom*

Supplemental Methods

Proteomic analysis

Somalogic's previously described standard processes for normalization, calibration, and quality control (QC) were followed.[13] The normalization and calibration factors are deemed acceptable as follows: hybridization control, intraplate median signal normalization and plate scale factors are expected to be between 0.4 and 2.5; the distribution of QC sample ratios is expected to have 85% of individual SOMAmer reagents in the total array between 0.8 and 1.2. SOMAmers outside these ranges were not considered for the current study. Moreover, SOMAmers with non-human and/or not-validated targets were excluded from further analyses. When multiple SOMAmers were present for the same target protein, those with the highest binding affinity were used. Thus, 4210 out of the total 5,284 modified aptamers were included in the current analyses. Individual sample quality was judged by comparing normalized median signal relative to the external reference standard, with the acceptable normalization scaling range being 0.4 - 2.5. Data from 1,066 samples of patients with HFrEF passed quality-control criteria.

Statistical analyses

Sex-based differences in prognostic value of serially measured circulating proteins were evaluated using time-dependent Cox models for each of the individual proteins. Values of the circulating proteins were estimated and extracted for the moments at which the proteins were actually measured by linear mixed effects (LME) modeling. Protein levels were used as dependent variables. Sex, sampling time during follow-up, and an interaction term for sex and sampling time were used as independent variable. To allow the protein trajectories to differ between individuals at baseline and over time, an intercept and slope were included in the random-effects design matrix. If the model was not significantly improved, only a random intercept was retained. Non-linear evolutions were tested using natural cubic splines for both the fixed- and random-effects parts of the LME model. If the model was not significantly improved, a linear evolution was retained. Thereupon, the individual temporal trajectories of all circulating proteins were estimated by

the LME models described above and values of the circulating proteins as estimated by the models, for the moments at which the proteins were actually measured, were extracted. Subsequently, the estimated protein levels were standardized and entered into single protein time-dependent Cox models.

Supplemental Tables

Supplemental Table S1. Follow-up and study endpoints.

Primary study endpoint comprised the composite of:	Patients that reached endpoint during follow-up	Patients that reached primary study endpoint*
Re-hospitalization for acute of worsened HF	18 women and 72 men	18 women and 72 men
LVAD placement	2 women and 11 men	1 woman and 5 men
Heart transplantation	4 women and 13 men	2 women and 8 men
Cardiovascular death	6 women and 27 men	2 women and 6 men

*Only the first event was used for analysis in patients who reached multiple endpoints during follow-up (39 out of 114). Abbreviations: HF, heart failure.

Supplemental Figures

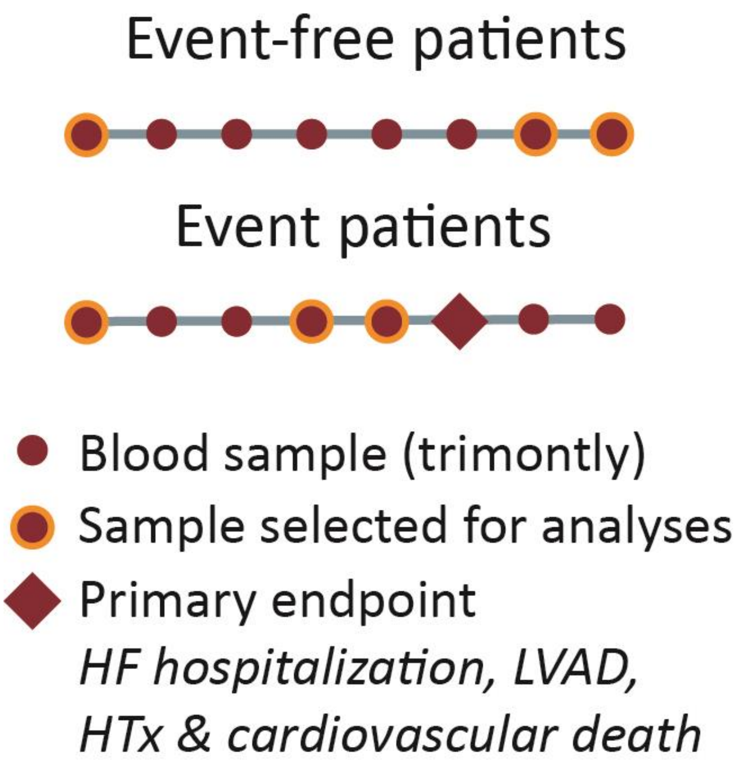

Supplemental Figure S1. Sample selection.

For the current investigation, all baseline blood samples were selected. Additionally, the last two samples drawn before the occurrence of the primary endpoint (PE), or the last two samples that were available before censoring for patients who remained endpoint-free, were selected.

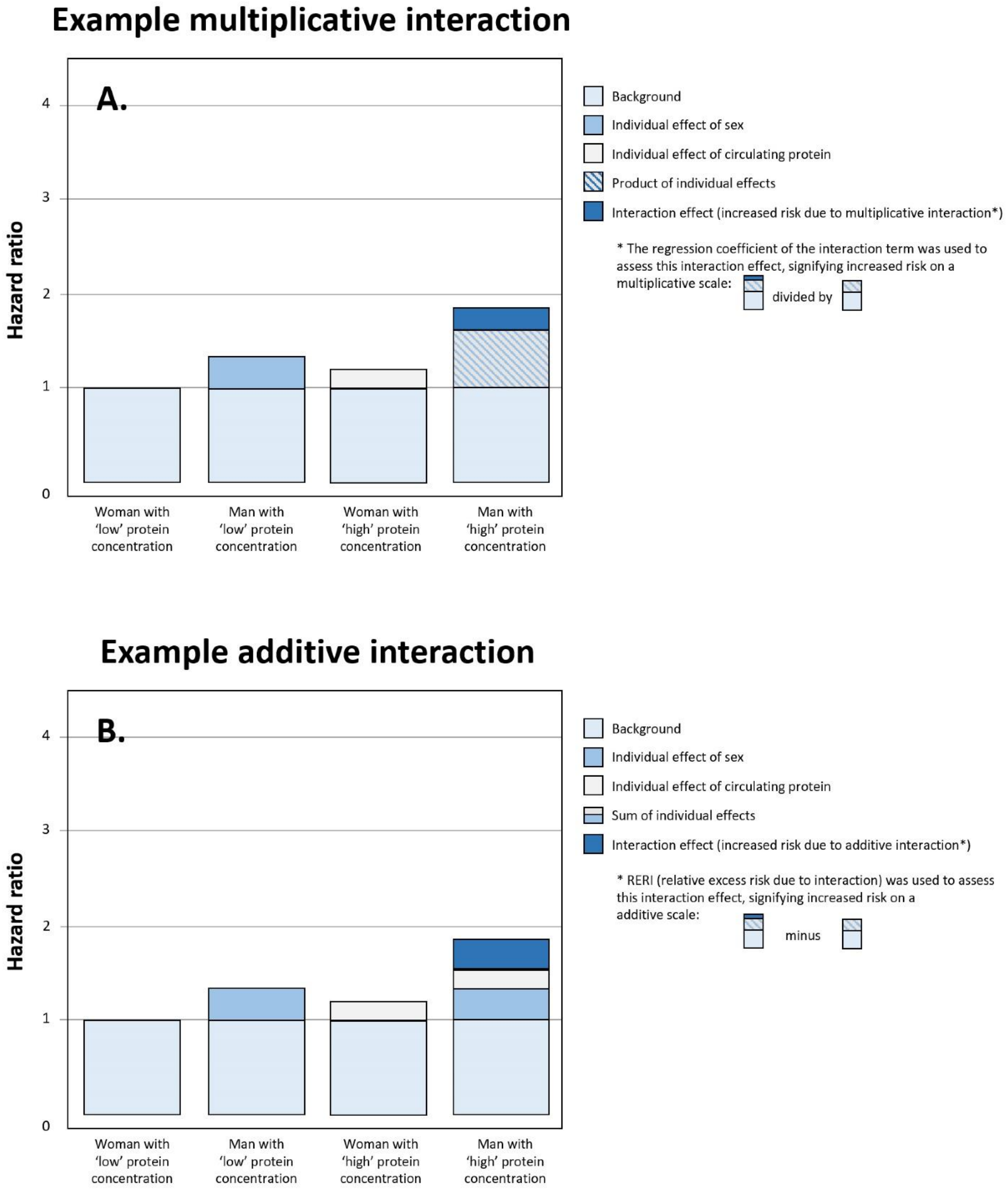

Supplemental Figure S2. Examples of interaction on the multiplicative and additive scale.

Interaction on the multiplicative scale (A) signifies that the combined effect of sex and protein level is larger [or smaller] than the product of the individual effects, and was assessed using the regression coefficient of the interaction term. Interaction on the additive scale (B) signifies that the combined effect of sex and protein level is larger [or smaller] than the sum of the individual effects, and was assessed using relative excess risk due to interaction (RERI).

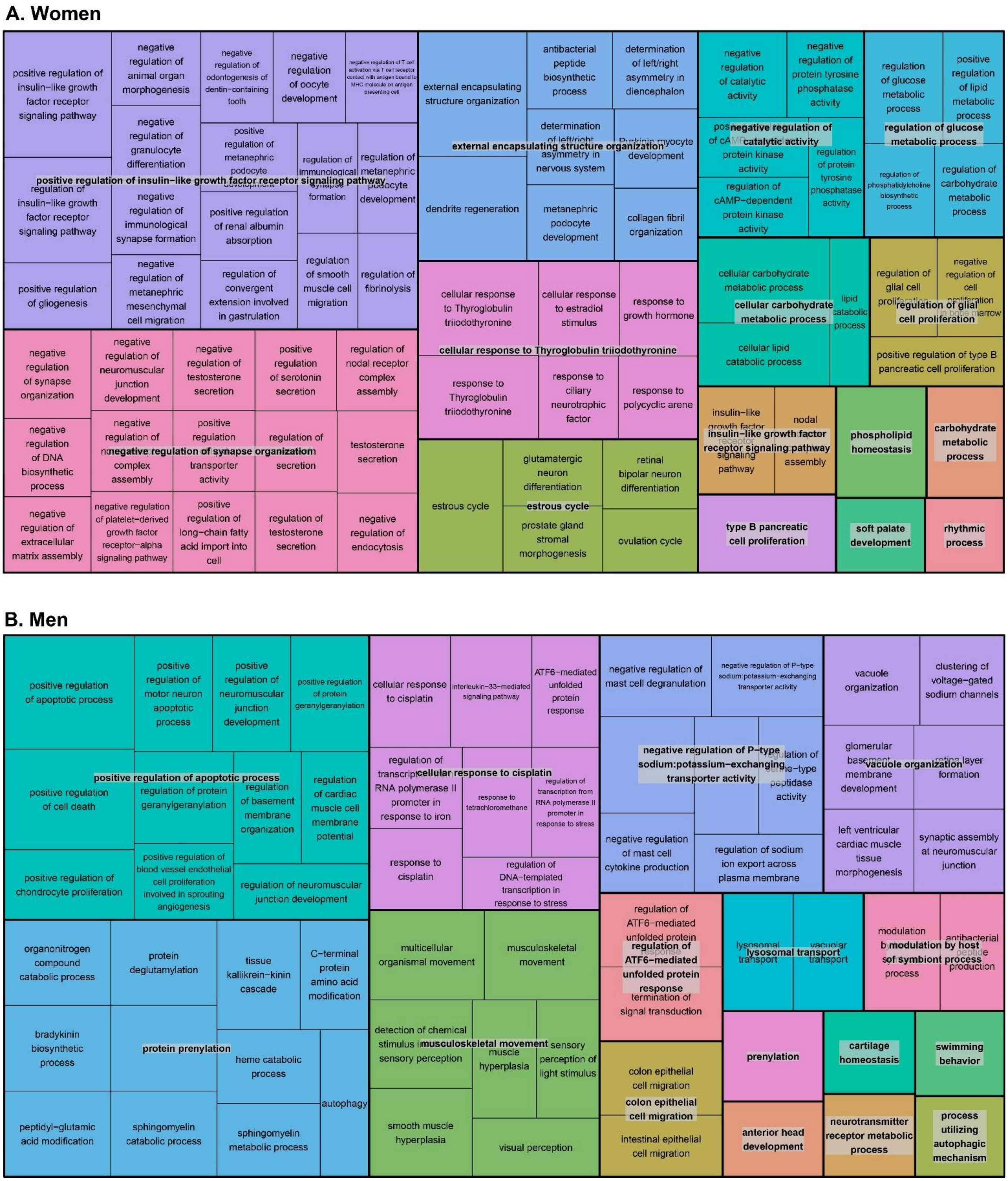

Supplemental Figure S3. Visualization of gene-enrichment analysis.

Treemap of GO biological processes associated with the circulating protein profile of women (A) and men (B), generated using REVIGO [38], a visualization tool that groups closely related GO terms together based on network analysis. Groups of closely related GO terms are plotted together in the same color. Size of the GO term blocks is proportional to the –log10(p-value) of their association with the sex-specific protein profile. Not all significant biological processes are represented as REVIGO removes some redundant terms that are very similar to other terms.

A1. Single protein unadjusted time−dependent Cox models

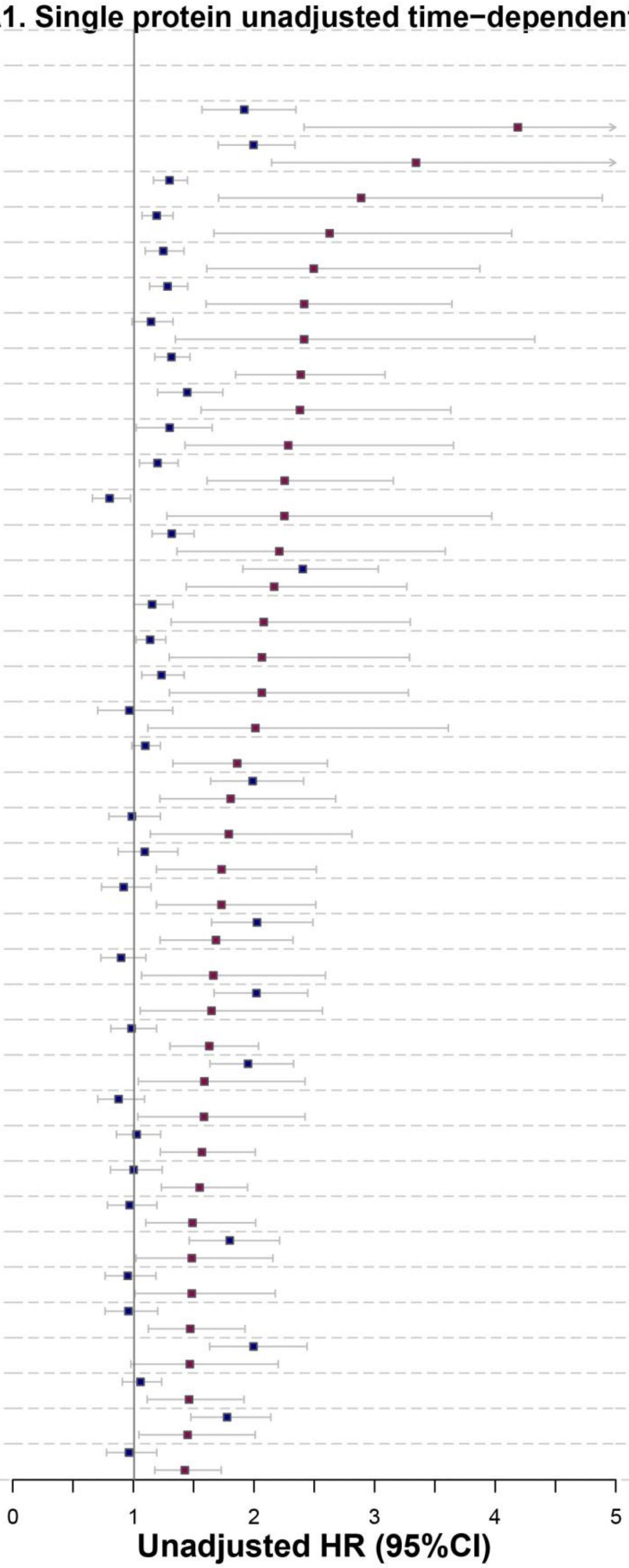

Proteins*	Multiplicative P−value	Additive P−value
PLAUR	0.009	0.363
FBLN5	0.032	0.121
GALNT1	0.004	0.056
NTF3	0.001	0.029
DDR2	0.003	0.053
PVRL2	0.004	0.05
SLIT2	0.015	0.098
BMP10	<0.001	0.004
FABP1	0.034	0.207
RNASE3	0.036	0.179
GLRX	0.001	0.016
IGFBP5	0.001	0.027
PARVA	0.044	0.208
FABP3	0.666	0.024
ADAMTS1	0.016	0.087
HGF	0.015	0.079
TNFRSF6B	0.037	0.146
ABL1	0.031	0.102
FURIN	0.004	0.033
GRN	0.666	0.049
IL16	0.02	0.075
COL15A1	0.039	0.151
DLK1	0.004	0.026
TRA2B	0.345	0.031
CLIC5	0.013	0.045
SAA1	0.407	0.03
HOPX	0.001	0.006
ENPP2	0.38	0.016
GLUL	0.015	0.045
MAPK11	0.007	0.033
PBRM1	0.006	0.037
INHBA.INHBC	0.021	0.061
TFF2	0.377	0.048
MEOX2	0.049	0.097
RBP4	0.017	0.054
ADIPOQ	0.183	0.014
NDRG1	0.043	0.118
CD14	0.289	0.048
IL22	0.008	0.027

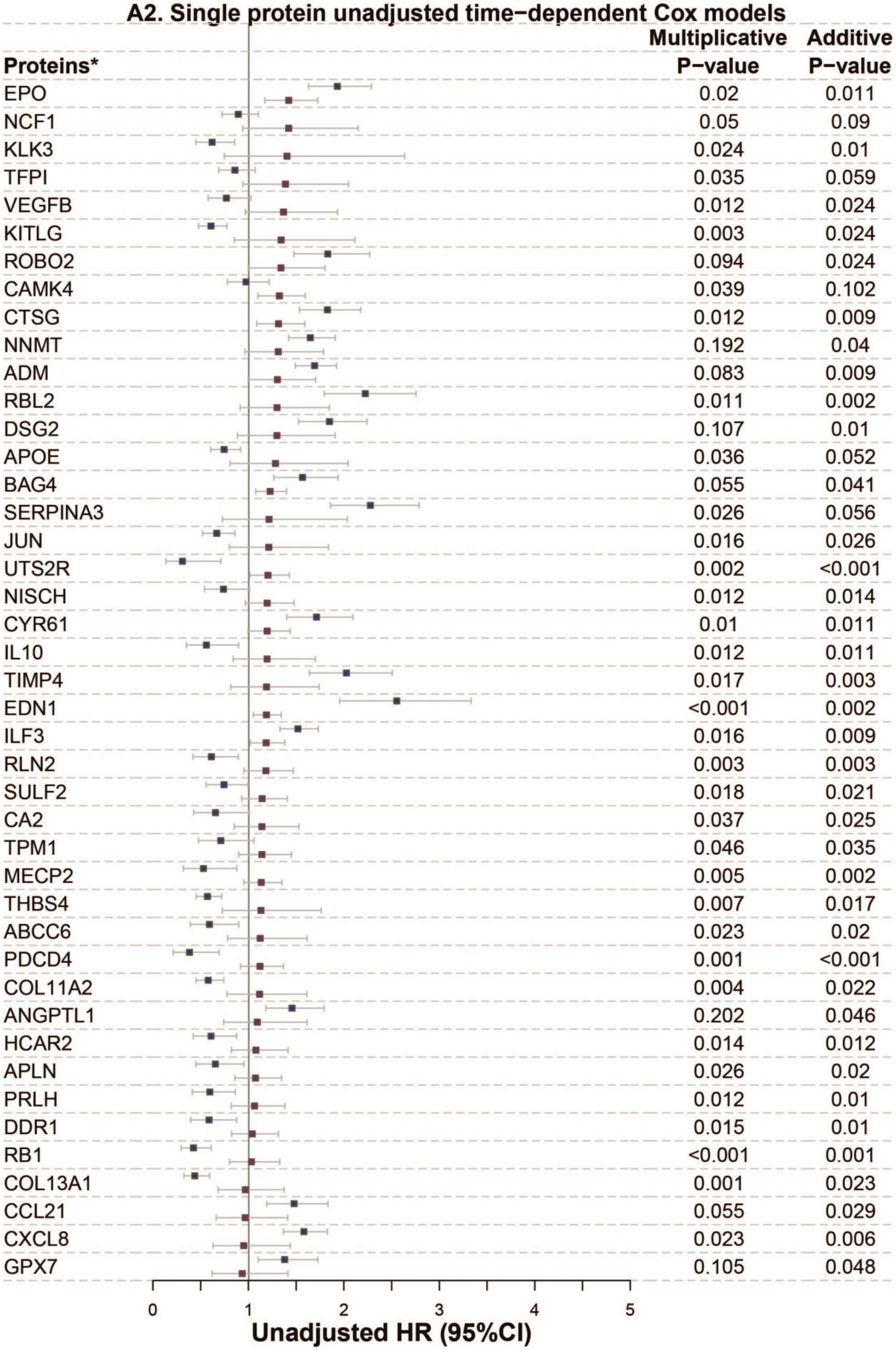
A2. Single protein unadjusted time−dependent Cox models
Proteins*
Multiplicative P−value
Additive P−value
EPO 0.02 0.011
NCF1 0.05 0.09
KLK3 0.024 0.01
TFPI 0.035 0.059
VEGFB 0.012 0.024
KITLG 0.003 0.024
ROBO2 0.094 0.024
CAMK4 0.039 0.102
CTSG 0.012 0.009
NNMT 0.192 0.04
ADM 0.083 0.009
RBL2 0.011 0.002
DSG2 0.107 0.01
APOE 0.036 0.052
BAG4 0.055 0.041
SERPINA3 0.026 0.056
JUN 0.016 0.026
UTS2R 0.002 <0.001
NISCH 0.012 0.014
CYR61 0.01 0.011
IL10 0.012 0.011
TIMP4 0.017 0.003
EDN1 <0.001 0.002
ILF3 0.016 0.009
RLN2 0.003 0.003
SULF2 0.018 0.021
CA2 0.037 0.025
TPM1 0.046 0.035
MECP2 0.005 0.002
THBS4 0.007 0.017
ABCC6 0.023 0.02
PDCD4 0.001 <0.001
COL11A2 0.004 0.022
ANGPTL1 0.202 0.046
HCAR2 0.014 0.012
APLN 0.026 0.02
PRLH 0.012 0.01
DDR1 0.015 0.01
RB1 <0.001 0.001
COL13A1 0.001 0.023
CCL21 0.055 0.029
CXCL8 0.023 0.006
GPX7 0.105 0.048
0
1
2
3
4
5
Unadjusted HR (95%CI)

A3. Single protein unadjusted time−dependent Cox models

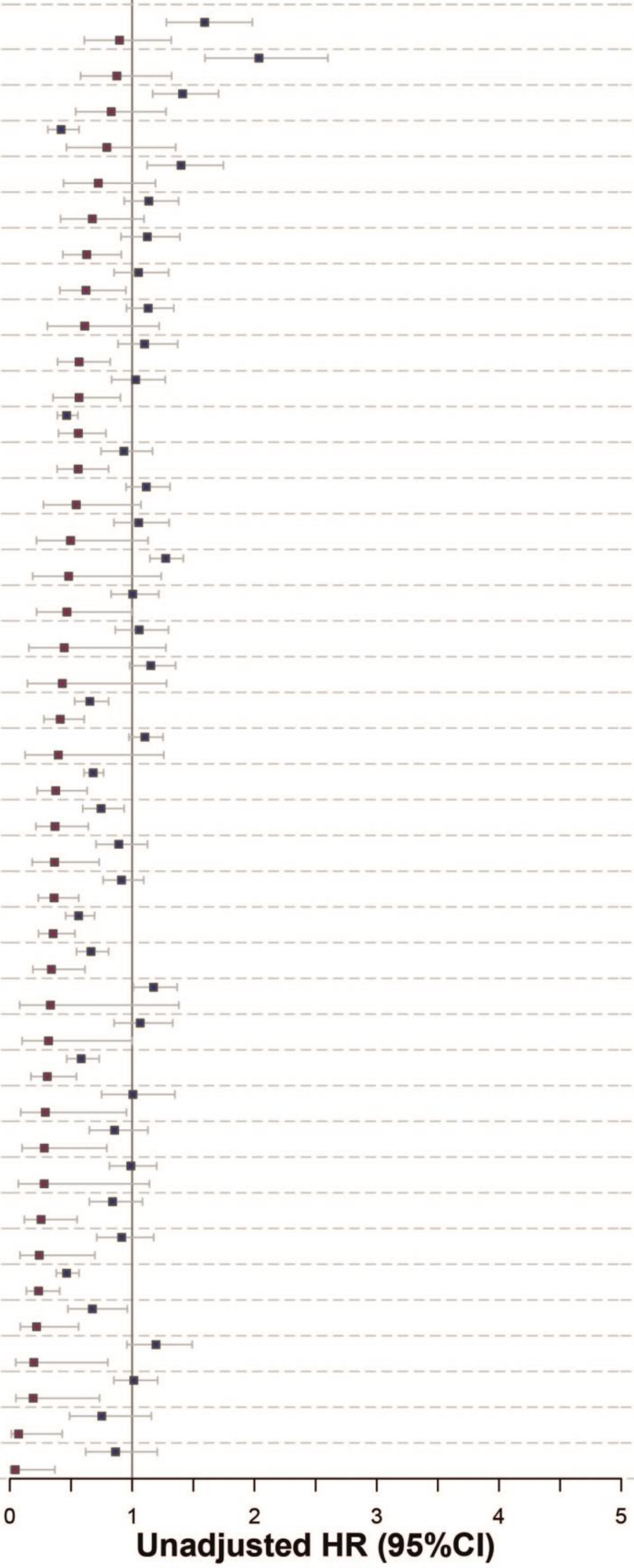

Proteins*	Multiplicative P−value	Additive P−value
CCT7	0.011	0.012
HS6ST2	0.001	0.002
SMPD1	0.027	0.021
SOCS3	0.042	0.032
PLXDC1	0.018	0.009
FCGR2B	0.051	0.041
FCGR2A	0.008	0.021
C5	0.029	0.043
GLA	0.091	0.036
HP	0.003	0.008
CTSA	0.023	0.057
FCN3	0.341	0.025
MET	0.02	0.102
RASA1	0.043	0.011
TNFRSF10A	0.083	0.048
SST	0.045	0.002
HIF1A	0.055	0.033
SPRED1	0.114	0.039
GZMB	0.080	0.013
KIT	0.041	0.96
TIAM1	0.084	0.007
APOA1	0.029	0.24
PTPRJ	0.021	0.215
CXCL5	0.017	0.041
C1GALT1C1	<0.001	0.002
EGFR	0.048	0.396
NOTCH1	0.035	0.375
ANXA5	0.084	0.004
SCN2B	0.042	0.006
HYAL1	0.041	0.618
TJP1	0.047	0.036
SIRT3	0.042	0.043
HNF1A	0.081	0.014
RAG1	0.004	0.101
RELB	0.016	0.027
PRCP	0.023	0.123
PPP2R3A	0.028	0.518
BCL6	0.013	0.001
EIF4G3	0.016	0.001
MYB	0.012	0.579
RGS4	0.007	0.206

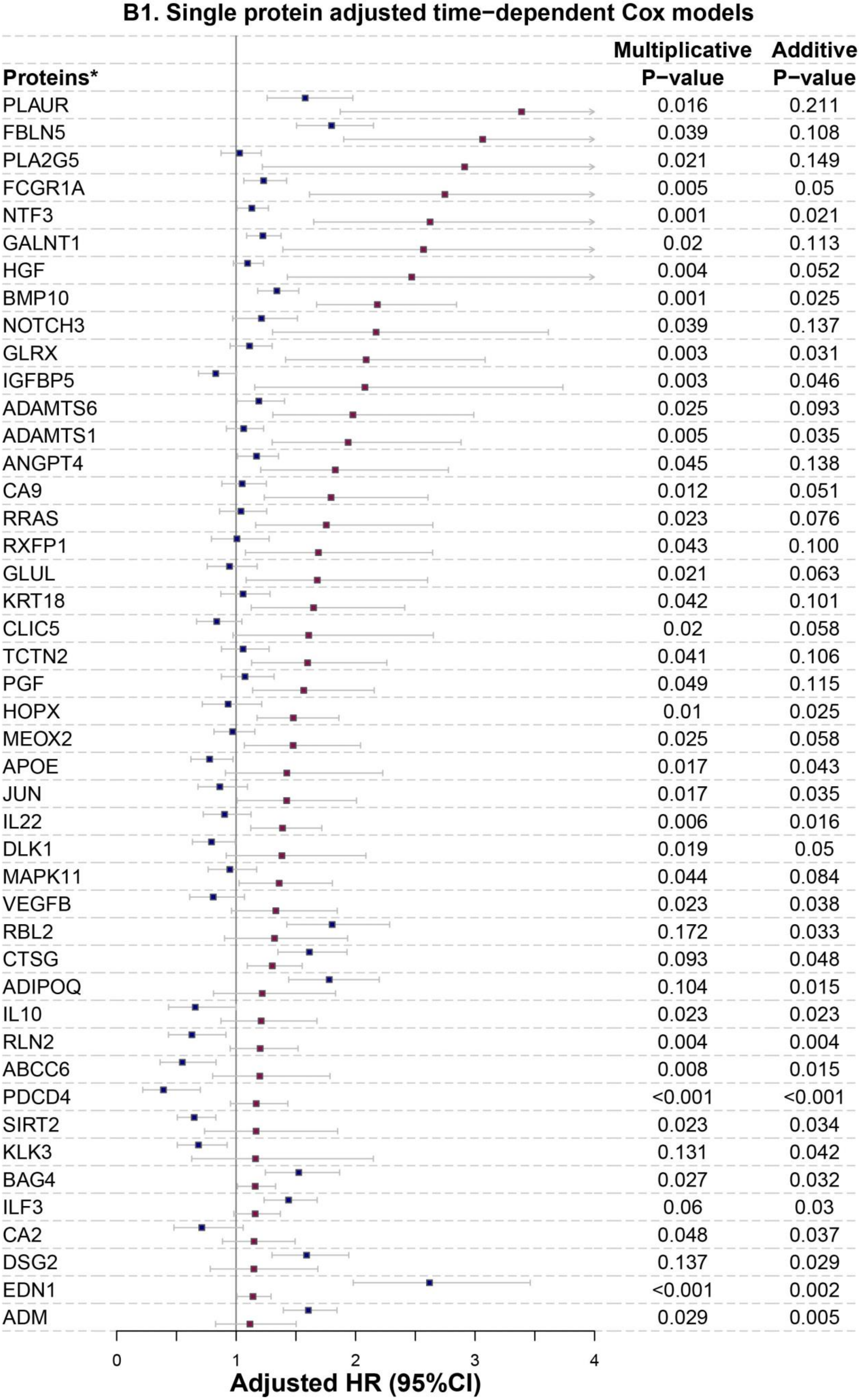
B1. Single protein adjusted time−dependent Cox models
Proteins*
Multiplicative P−value
Additive P−value
PLAUR 0.016 0.211
FBLN5 0.039 0.108
PLA2G5 0.021 0.149
FCGR1A 0.005 0.05
NTF3 0.001 0.021
GALNT1 0.02 0.113
HGF 0.004 0.052
BMP10 0.001 0.025
NOTCH3 0.039 0.137
GLRX 0.003 0.031
IGFBP5 0.003 0.046
ADAMTS6 0.025 0.093
ADAMTS1 0.005 0.035
ANGPT4 0.045 0.138
CA9 0.012 0.051
RRAS 0.023 0.076
RXFP1 0.043 0.100
GLUL 0.021 0.063
KRT18 0.042 0.101
CLIC5 0.02 0.058
TCTN2 0.041 0.106
PGF 0.049 0.115
HOPX 0.01 0.025
MEOX2 0.025 0.058
APOE 0.017 0.043
JUN 0.017 0.035
IL22 0.006 0.016
DLK1 0.019 0.05
MAPK11 0.044 0.084
VEGFB 0.023 0.038
RBL2 0.172 0.033
CTSG 0.093 0.048
ADIPOQ 0.104 0.015
IL10 0.023 0.023
RLN2 0.004 0.004
ABCC6 0.008 0.015
PDCD4 <0.001 <0.001
SIRT2 0.023 0.034
KLK3 0.131 0.042
BAG4 0.027 0.032
ILF3 0.06 0.03
CA2 0.048 0.037
DSG2 0.137 0.029
EDN1 <0.001 0.002
ADM 0.029 0.005
0
1
2
3
4
Adjusted HR (95%CI)

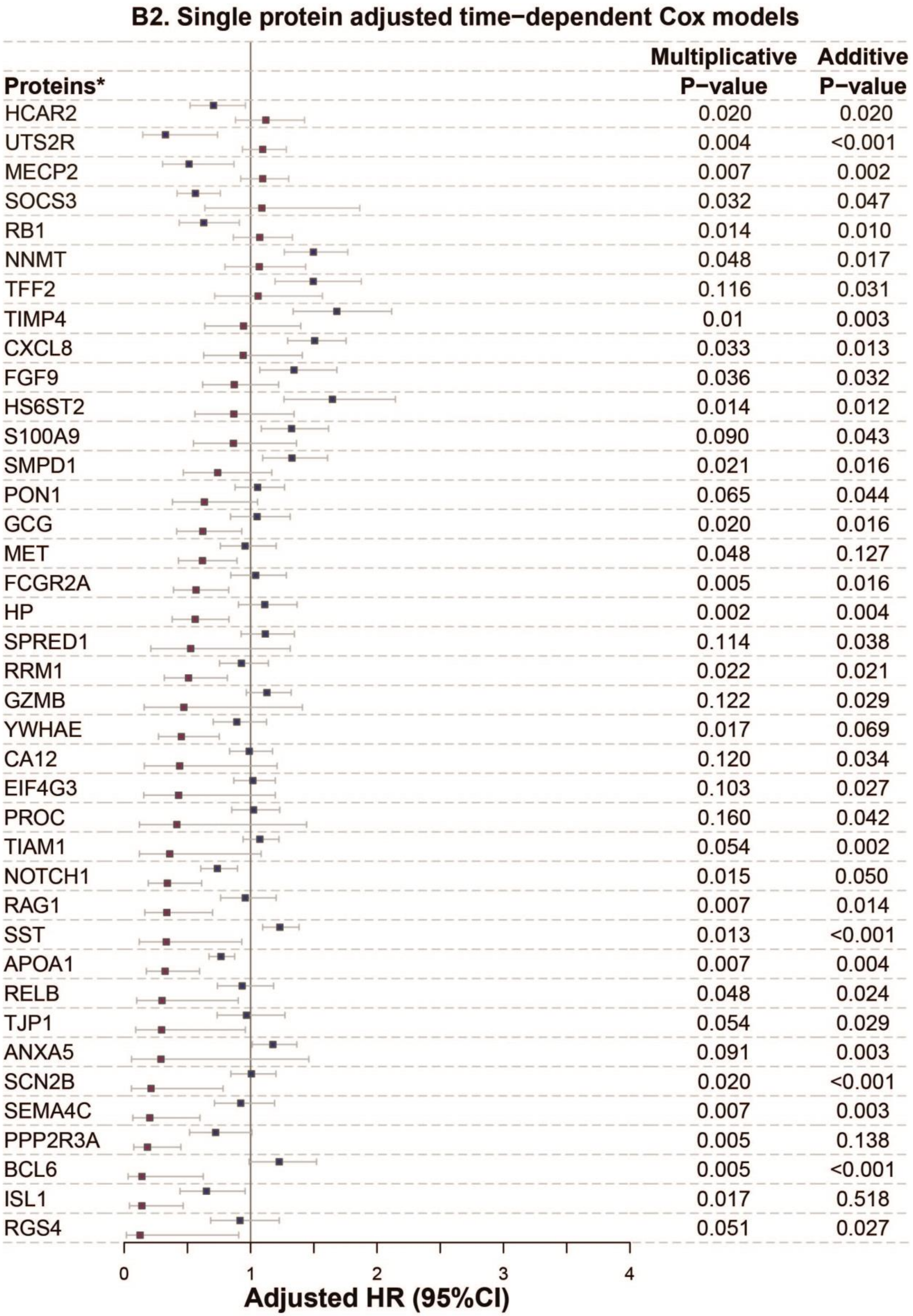

Supplemental Figure S4. Sex-based differences in predictive value of serially measured proteins (without multiple testing correction).

* Proteins are depicted by their HUGO Gene Nomenclature Committee (HGNC) gene symbol.
